# Green tea-derived theabrownin induces cellular senescence and apoptosis of hepatocellular carcinoma through p53 signaling activation and bypassed JNK signaling suppression

**DOI:** 10.1186/s12935-022-02468-3

**Published:** 2022-01-25

**Authors:** Jiaan Xu, Xiujuan Xiao, Bo Yan, Qiang Yuan, Xiaoqiao Dong, Quan Du, Jin Zhang, Letian Shan, Zhishan Ding, Li Zhou, Thomas Efferth

**Affiliations:** 1 College of Pharmaceutical Sciences, Zhejiang Chinese Medical University, Hangzhou, 310053 China; 2 The First Affiliated Hospital, Zhejiang Chinese Medical University, Hangzhou, 310053 China; 3 Department of Neurosurgery, Affiliated Hangzhou First People’s Hospital, Zhejiang University School of Medicine, Hangzhou, 310006 China; 4 Theabio Co., Ltd, Hangzhou, 310000 China; 5 Cell Resource Bank and Integrated Cell Preparation Center of Xiaoshan District, Hangzhou Regional Cell Preparation Center (Shangyu Biotechnology Co., Ltd), Hangzhou, China; 6 College of Medical Technology, Zhejiang Chinese Medical University, Hangzhou, 310053 China; 7 Department of Pharmaceutical Biology, Institute of Pharmacy and Biochemistry, Johannes Gutenberg University, Mainz, Germany

**Keywords:** Theabrownin, Hepatocellular carcinoma, Cellular senescence, Apoptosis, p53, JNK, SK-Hep-1

## Abstract

**Background:**

Theabrownin (TB) is a bioactive component of tea and has been reported to exert effects against many human cancers, but its efficacy and mechanism on hepatocellular carcinoma (HCC) with different p53 genotypes remains unclarified.

**Methods:**

MTT assay, DAPI staining, flow cytometry and SA-β-gal staining were applied to evaluate the effects of TB on HCC cells. Quantitative real time PCR (qPCR) and Western blot (WB) were conducted to explore the molecular mechanism of TB. A xenograft model of zebrafish was established to evaluate the anti-tumor effect of TB.

**Results:**

MTT assays showed that TB significantly inhibited the proliferation of SK-Hep-1, HepG2, and Huh7 cells in a dose-dependent manner, of which SK-Hep-1 was the most sensitive one with the lowest IC_50_ values. The animal data showed that TB remarkably suppressed SK-Hep-1 tumor growth in xenograft model of zebrafish. The cellular data showed TB's pro-apoptotic and pro-senescent effect on SK-Hep-1 cells. The molecular results revealed the mechanism of TB that p53 signaling pathway (p-ATM, p-ATR, γ-H2AX, p-Chk2, and p-p53) was activated with up-regulation of downstream senescent genes (*P16*, *P21*, *IL-6* and *IL-8*) as well as apoptotic genes (*Bim*, *Bax* and *PUMA*) and proteins (Bax, c-Casp9 and c-PARP). The p53-mediated mechanism was verified by using p53-siRNA. Moreover, by using JNK-siRNA, we found JNK as a bypass regulator in TB's mechanism.

**Conclusions:**

To sum up, TB exerted tumor-inhibitory, pro-senescent and pro-apoptotic effects on SK-Hep-1 cells through ATM-Chk2-p53 signaling axis in accompany with JNK bypass regulation. This is the first report on the pro-senescent effect and multi-target (p53 and JNK) mechanism of TB on HCC cells, providing new insights into the underlying mechanisms of TB's anti-HCC efficacy.

## Introduction

Liver cancer is a major contributor to the world’s cancer burden, with more than 800,000 new cases and 700,000 deaths each year [1]. Hepatocellular carcinoma (HCC), a principal histologic type of liver cancer, represents more than 75% of primary liver cancers [2]. Due to the aging and population growth, the global incidence of HCC increased by 75% between 1990 and 2015, with the highest incident, mortality and years of life lost in east Asia [3]. The worldwide risk factor of HCC is heterogeneous. Hepatitis B virus (HBV) is the leading cause of incident cases of HCC in Africa and East Asia, while alcoholic liver disease (ALD) and hepatitis C (HCV) are the most common risk factor for HCC in the USA [4]. Prognosis of HCC is poor all around the world, resulting in a rough equivalent of incidence and mortality rates [5]. For early-stage HCC, radiofrequency ablation or surgical resection remains the main treatment. However, up to 75% of patients undergoing surgery experience recurrence within 5 years [6]. Over the last decade, targeted therapy (sorafenib) has become the major systemic strategy which can significantly improve the overall survival for patients with unresectable HCC [7]. However, the adverse effects of sorafenib, such as diarrhea, hypertension, and hand-foot skin reaction (HFSR), as well as its low bioavailability limit its clinical application [8, 9]. Therefore, there is an urgent need to explore potential drug candidates for HCC.

Alike many carcinomas, HCC has multiple genomic mutations. The prevalent mutations locate at TERT promoters, such as *TP53*, *CTNNB1*, *AXIN1* and *CDKN2A* [10, 11]. In most cases of HCC (> 90%), telomerase activation, relating to TERT promoter mutations, is necessary for malignant transformation and tumor progression [12, 13]. Of these, *CTNNB1* mutations frequently activate the Wnt/β-catenin pathway, particularly in patients with HBV uninfection and well-differentiated tumors (11–37% of HCC cases) [14, 15]. By contrast, inactivation of p53 caused by *TP53* mutations particularly appears in cases related to HBV infection and aflatoxin B1 exposure [16–18]. As a tumor suppressor, *TP53* encodes p53 transcriptional factor to prevent tumor development through permanently suppression of cell proliferation by cell cycle arrest and facilitation of cell death by apoptosis [19]. However, *TP53* mutation or deletion occurs in nearly a half of human cancers, while tumors carrying wild-type *TP53* usually get rid of the p53 defense mechanism via interaction with negative regulators, such as MDM2 and MDM4 [20, 21]. Thus, reactivation of p53 becomes a potential strategy for cancer treatment [22–25] For instance, a p53-MDM2 inhibitor, RG7388, activates p53 signaling pathway by selectively blocking p53-MDM2 binding, exhibiting encouraging anti-cancer efficacy in several different clinical trials [26, 27].

Green tea, derived from leaves of *Camellia sinensis*, was originally used as medicine in ancient China. Meanwhile, it was one of the most prevalent beverages worldwide for centuries. In recent decades, green tea’s health benefits have been extensively studied, including anti-inflammation, cardiovascular-protection, anti-obesity, anti-cancer, and hepato-protection [28–32]. A large prospective cohort study on 164,681 adult Chinese men has concluded that 10 g or more green tea consumption per day decreased the mortality from cancers, indicating an anti-cancer efficacy of green tea [33]. As the main pigment of tea, theabrownin (TB) is a heterogeneous polymer (3–50 kDa) with high water solubility and possesses regulatory effects in improving metabolism of glucose and serum lipids [34–36]. Our previous studies have discovered TB’s pro-apoptotic effects on human carcinoma cells and sarcoma cells both through a p53-mediated mechanism [37, 38]. To date, the efficacy of TB on human HCC cells with different p53 genotypes remains unclear. To fill this gap, this study adopted HCC cell lines, including SK-Hep-1 (p53-WT), HepG2 (p53-WT) and Huh7 (p53-mut), to evaluate TBʹs effects and the p53-related mechanism.

## Materials and methods

### Chemicals and reagents

Theabrownin (> 90% of purity) was supplied by Theabio Co., Ltd (Hangzhou, China) (Batch number: 20181210001) and dissolved in the corresponding culture medium. Cis-platin (S116614) was purchased from Selleck (Shanghai, China) and dissolved in 50 °C warm water to 3 mg/ml and then diluted in cultured water to 15 µg/ml, according to the instruction. Fetal bovine serum (FBS), 0.25% trypsin Dulbecco’s modified eagle medium (DMEM) and Minimum Essential Medium α (MEM α), were obtained from Thermo Fisher Scientific (MA, USA). Annexin-V/FITC apoptosis detection kit was purchased from BD Biosciences (NJ, USA). Senescence β-galactosidase (SA-β-gal) staining kit was purchased from Beyotime (Shanghai, China). All antibodies were obtained from Cell Signaling Technology (MA, USA).

### Cell culture

The human HCC SK-Hep-1, HepG2, and Huh7 cell lines were purchased from Cell Bank of Chinese Academy of Sciences, China. SK-Hep-1 cells were cultured in MEM α containing 10% FBS at 37 °C and 5% CO_2_. Cells were passaged two or three times per week. HepG2 and Huh7 cells were cultured in DMEM containing 10% FBS at 37 °C and 5% CO_2_. Cells were passaged two or three times per week.

### Zebrafish

Wild-type AB strain of zebrafish was purchased from the China Zebrafish Resource Center, Institute of Hydrobiology, China Academy of Science (Wuhan, China) and accredited by the Association for Assessment and Accreditation of Laboratory Animal Care International (SYXK 2012-0171). Larval zebrafish (2 dpf, days post fertilization) were obtained by natural pair-mating and raised at 28 °C under a 10/14 h dark/light cycle.

### Cell viability assay and morphological observations

Cells viability was determined by MTT assays. Briefly, cells (8 × 10^3^ cells/well) were seeded in 96-well plates overnight, followed by exposure to various concentrations of TB for 24 or 48 h. All wells were added ten microliters of MTT reagent (5.0 mg/ml) and inoculated for 4 h. Afterwards, DMSO was added to dissolve the formazan crystals. Optical density (OD) values at 490 nm were detected by a microplate reader (Bio-Rad, USA). Inhibitory rate (%) = [1 − (OD_TB_/OD_control_)] × 100. The 50% inhibitory concentrations (IC_50_) of 24 and 48 h were calculated by SPSS Software. And the IC_25_, IC_50_, and IC_75_ were chosen as the low, medium and high doses of TB for further use.

### Animal experiment in zebrafish

To determine the dose range of TB, larval zebrafish (3 dpf) were treated with various concentrations of TB (0, 6.25, 12.5, 25, 50, 100, 250, 500, 1000, and 2000 µg/ml) for 24 h, followed by the observation under a stereoscopic microscope. According to the preliminary studies, no observed adverse effect level (NOAEL) of TB was estimated.

Each larval zebrafish (2 dpf) was microinjected 200 SK-Hep-1 cells labeled with CM-Dil (red fluorescence) into the yolk sac as previously described [37]. After 24 h, 30 larval/group were selected under fluorescence microscope and randomly cultured in 6‐well plates. The model group, Cis-platin (15 µg/ml) group, and TB (1.7, 5.6, and 16.7 µg/ml) group were set up. Zebrafish of each group were observed under fluorescence microscope after 24 h treatment. The fluorescence intensity (FI) of SK-Hep-1 xenograft tumor was calculated by Image pro plus 6.0 software. The inhibitory rate (IF) was calculated as: Inhibitory rate (%) = [1 − (IF_treated_/IF_untreated_)] × 100%.

### DAPI staining

The apoptosis of TB-treated SK-Hep-1 cells was detected using DAPI staining. Briefly, SK-Hep-1 cells were treated with TB at 50, 100, and 150 µg/ml for 24 h respectively. Cells were fixed with 4% paraformaldehyde for 15 min and then stained with DAPI (Invitrogen, USA) for 4 min in dark. The apoptotic nuclei of cells (bright blue nuclei and condensed chromatin with apoptotic bodies) were observed under a fluorescence microscope.

### Flow cytometry

The apoptosis of TB-treated SK-Hep-1 cells was also detected using an Annexin-V/PI apoptosis kit. Briefly, TB-treated cells were collected and then labeled with FITC Annexin V and PI according to the manufacturer’s protocol. Fluorescence intensity of the cells was measured by a flow cytometry (BD Biosciences, USA). The analysis was replicated thrice and the values of upper right and lower right quadrants of the flow cytometric dot plot were summed to calculate the apoptosis rate (%) for each TB treatment.

### SA-β-Gal assay

SK-Hep-1 cells were treated with TB at 50 and 100 µg/ml for 48 h. Senescence of SK-Hep-1 cells was evaluated by SA-β-gal assay according to the instructions. Briefly, the cells were washed twice with PBS, and then incubated in fixing solution (4% paraformaldehyde in PBS) at room temperature for 15 min. Subsequently, cells were stained with working solution (1 ml working solution contained 10 µl staining solution A, 10 µl staining solution B, 930 µl staining solution C, and 50 µl X-Gal solution) for 24 h. To quantify the percentage of SA-β-gal-positive cells, five digital images were randomly captured by a microscope and the positive cells from each group were counted.

### Quantitative real time PCR (qPCR) assay

As previously described [38], total RNA was extracted using TRIzol reagent and quantified by NanoDrop2000 spectrophotometer (Thermo, USA). Then the RNA was reverse transcribed to cDNA using Primescript RT master mix (TaKaRa, Japan). According to the instructions, mRNA quantity was determined by RT-qPCR system (Applied Biosystems, USA) with SYBR Premix Ex Taq II (Tli RnaseH Plus). The relative mRNA expressions were analyzed by 2^−ΔΔCT^ method and *β-Actin* was used as the reference gene. Primer sequences are shown in Table 1.

**Table 1:** Primer sequences used for qPCR analysis and siRNA sequences

Gene	Forward primer	Reverse primer
*β-actin*	5′-CCCGCGAGTACAACCTTCT-3′	5′-CGTCATCCATGGCGAACT-3′
*PUMA*	5′-GACCTCAACGCACAGTACGAG-3′	5′-AGGAGTCCCATGATGAGATTGT-3′
*P53*	5′-TCAACAAGATGTTTTGCCAACTG-3′	5′-ATGTGCTGTGACTGCTTGTAGATG-3′
*P21*	5′-GGCAGACCAGCATGACAGATT-3′	5′-GCGGATTAGGGCTTCCTCT-3′
*P16*	5′-CATGGTGCGCAGGTTCTTG-3′	5′-CGGGATGTGAACCACGAAA-3′
*JNK1*	5′-CCAGGACTGCAGGAACGAGT-3′	5′-CCACGTTTTCCTTGTAGCCC-3′
*JNK2*	5′-ATGACCCCTTACGTGGTGACA-3′	5′-CATGATGCAACCCACTGACC-3′
*IL-8*	5′-GAATGGGTTTGCTAGAATGTGATA-3′	5′-CAGACTAGGGTTGCCAGATTTAAC-3′
*IL-6*	5′-TCTCCACAAGCGCCTTCG-3′	5′-CTCAGGGCTGAGATGCCG-3′
*GADD45α*	5′-GAGAGCAGAAGACCGAAAGGA-3′	5′-CACAACACCACGTTATCGGG-3′
*Bax*	5′-CCTTTTCTACTTTGCCAGCAAAC-3′	5′-GAGGCCGTCCCAACCAC-3′
*Bim*	5′-ACCAAACCAAAGCCGTCATCA-3′	5′-GGAGCCAGTAAACGTATTGGAAG-3′
*siP53* ^*1*^	5′-GACUCCAGUGGUAAUCUAC‐3′	5′-GTAGATTACCACTGGAGTC-3′
*siP53* ^*2*^	5′-GUAGAUUACCACUGGAGUC‐3′	5′-GACTCCAGTGGTAATCTAC-3′
*siJNK1*	5′-GGGCCUACAGAGAGCUAGUUCUUAU-3′	5′-ATAAGAACTAGCTCTCTGTAGGCCC-3′
*siJNK2*	5′-CATGAAAGAATGTCCTACCTTCTTT-3′	5′-AGAAGGTAGGACATTCTTTCATGTT-3′

### Western blot (WB) analysis

As previously described [37], total proteins was extracted using RIPA lysis buffer with proteinase inhibitor cocktail (Bimake, USA). And the concentrations of protein were estimated using a Bradford assay kit (Thermo, USA). The proteins were separated by 8–12% SDS-PAGE and transferred onto a nitrocellulose membrane (Sartorius Stedim, Germany). The membrane was blocked with 5% BSA at 4 °C for 2 h, and subsequently incubated at 4 °C overnight with the following primary antibodies: β-actin, ATR, phospho-ATR, ATM, phospho-ATM, Chk2, phospho-Chk2, p53, phospho-p53, p21, γ-H2AX, PARP, c-Casp9, Bax and Bcl-2. After incubation with anti-rabbit or anti-mouse IgG HRP-conjugated antibody, all bands were detected using Western Lightning^®^ Plus ECL (Perkin Elmer, USA). And the results were visualized using X-ray film (Kodak, Japan).

### Small interfering RNA (siRNA) transfection treatment

Human p53-targeted (p53-siRNA), JNK1-targeted (JNK1-siRNA), JNK2-targeted (JNK2-siRNA) and negative control (NC-siRNA) siRNAs were designed by GenePharma (Shanghai, China). siRNA transfection was performed on SK-Hep-1 cells using Lipofectamine 2000 (Thermo, USA) according to the manufacturer’s instruction. Transfection efficacy was determined via qPCR and WB assays. The siRNA targeting sequences of *P53*, *JNK1*, and *JNK2* were shown in Table 1.

### Statistical analysis

The data analyses were performed using SPSS statistics software and were expressed as the mean ± standard deviation (SD). Statistical significance among different groups were examined using one-way ANOVA followed by Fisher’s least significant difference (LSD) comparison. *p* < 0.05 was considered statistically significant.

## Results

### Anti-proliferative effect of TB

**Fig. 1:**
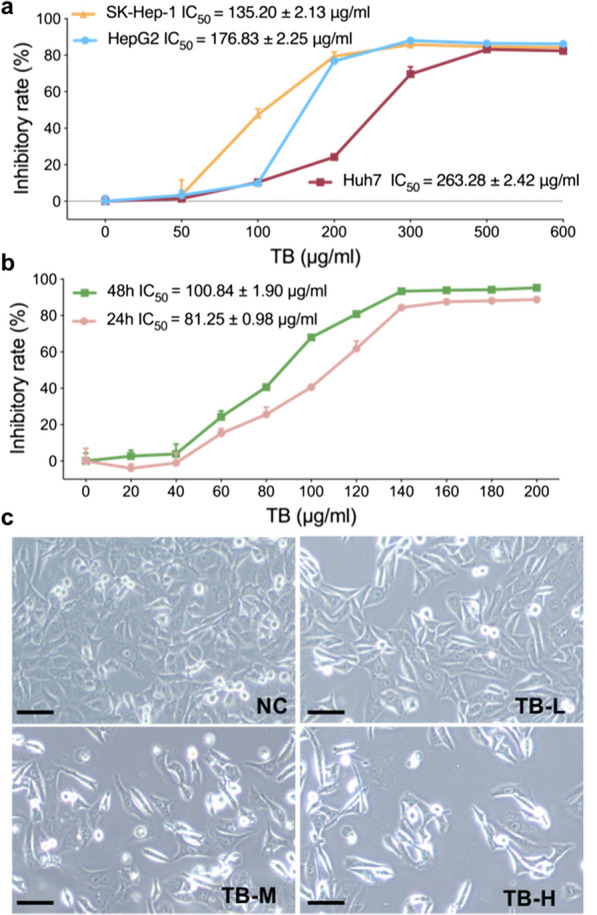
**a** Inhibitory rate of TB on SK-Hep-1, HepG2, and Huh7 cells at 24 h. **b** IC_50_ of TB on SK-Hep-1 cells at 24 and 48 h. **c** Morphological observation (light microscope) of SK-Hep1 cells with TB treatment for 24 h. Scale bar: 100 μm

Anti-proliferative effect of TB on HCC cell lines was determined by cell viability assays and cell morphological observation. TB inhibited proliferation of SK-Hep-1, HepG2 and Huh7 cells from 50 to 600 µg/ml (Fig. 1a). Given that the inhibitory effect on SK-Hep-1 cells was the strongest, SK-Hep-1 cells were selected for further assays. As depicted in Fig. 1b, TB significantly inhibited cell viability of SK-Hep-1 with the increasing dose and exposure time. And the IC_50_ values of 24 and 48 h were 100.84 ± 1.90 µg/ml and 81.25 ± 0.98 µg/ml. According to the IC_50_ values of 24 h, TB at 50, 100 and 150 µg/ml were selected as low, medium and high doses for in vitro assays. Microscopic analyses showed that TB treatment decreased the number of SK-Hep-1 cells and increased the number of the round cells and shrunken cells (Fig. 1c).

### Anti-tumor effect of TB in vivo

**Fig. 2:**
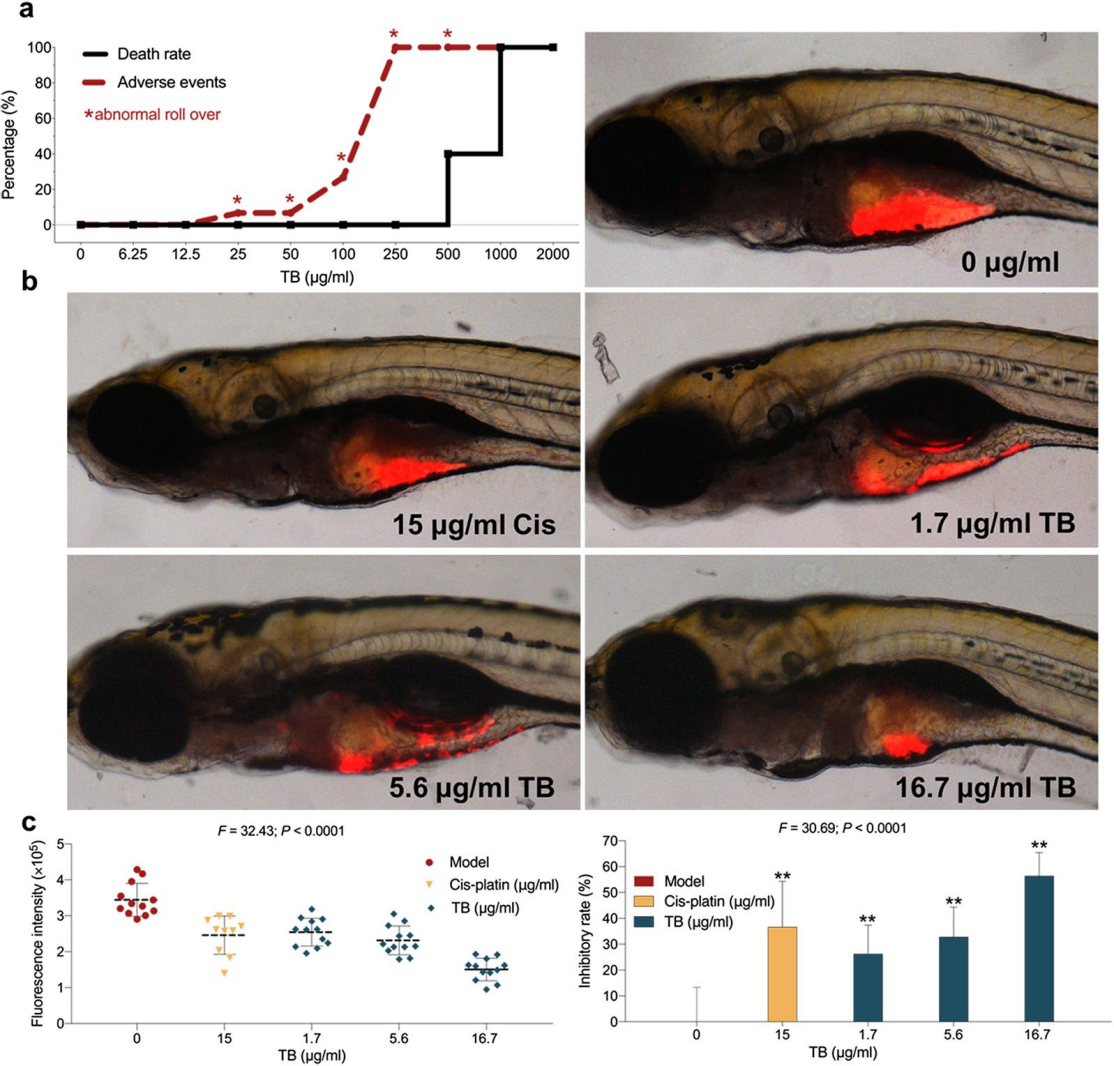
**a** The mortality and adverse events of larval zebrafish with TB treatment. **b** Observation of SK-Hep-1 xenograft zebrafish with Cis-platin or TB treatment. **c** Data of fluorescence intensity and inhibitory effect of Cis-platin or TB. The fluorescent area (red) represents the HCC tumor mass. Values were presented as the mean ± SD (n = 30). **p < 0.01 vs. model (0 µg/ml)

The zebrafish mortality and adverse events caused by TB were shown in Fig. 2a. TB obviously induced the adverse events (abnormal roll over) of zebrafish from 25 to 500 µg/ml. And the first fish death was observed at 500 µg/ml of TB, and all fishes were dead at 1000 µg/ml. Based on regression curve, TB’s NOAEL was calculated as 16.7 µg/ml. And 1.7, 5.6 and 16.7 µg/ml were selected as low, medium and high doses for in vivo experiment. As depicted in Fig. 2b, the HCC xenograft model was established successfully in zebrafish and TB dose-dependently suppressed SK-Hep-1 tumor growth. The inhibitory rates of TB at 1.7, 5.6 and 16.7 µg/ml were 26.15%, 32.78% and 56.30%, respectively. Cis-platin, one of the first-line of anticancer drugs, was applied as a positive control group. As depicted in Fig. 2c, high dose of TB (16.7 µg/ml) exerted comparable tumor-inhibitory effect to Cis-platin (15 µg/ml) in the zebrafish model within a short period.

### Pro-apoptotic effect of TB

**Fig. 3:**
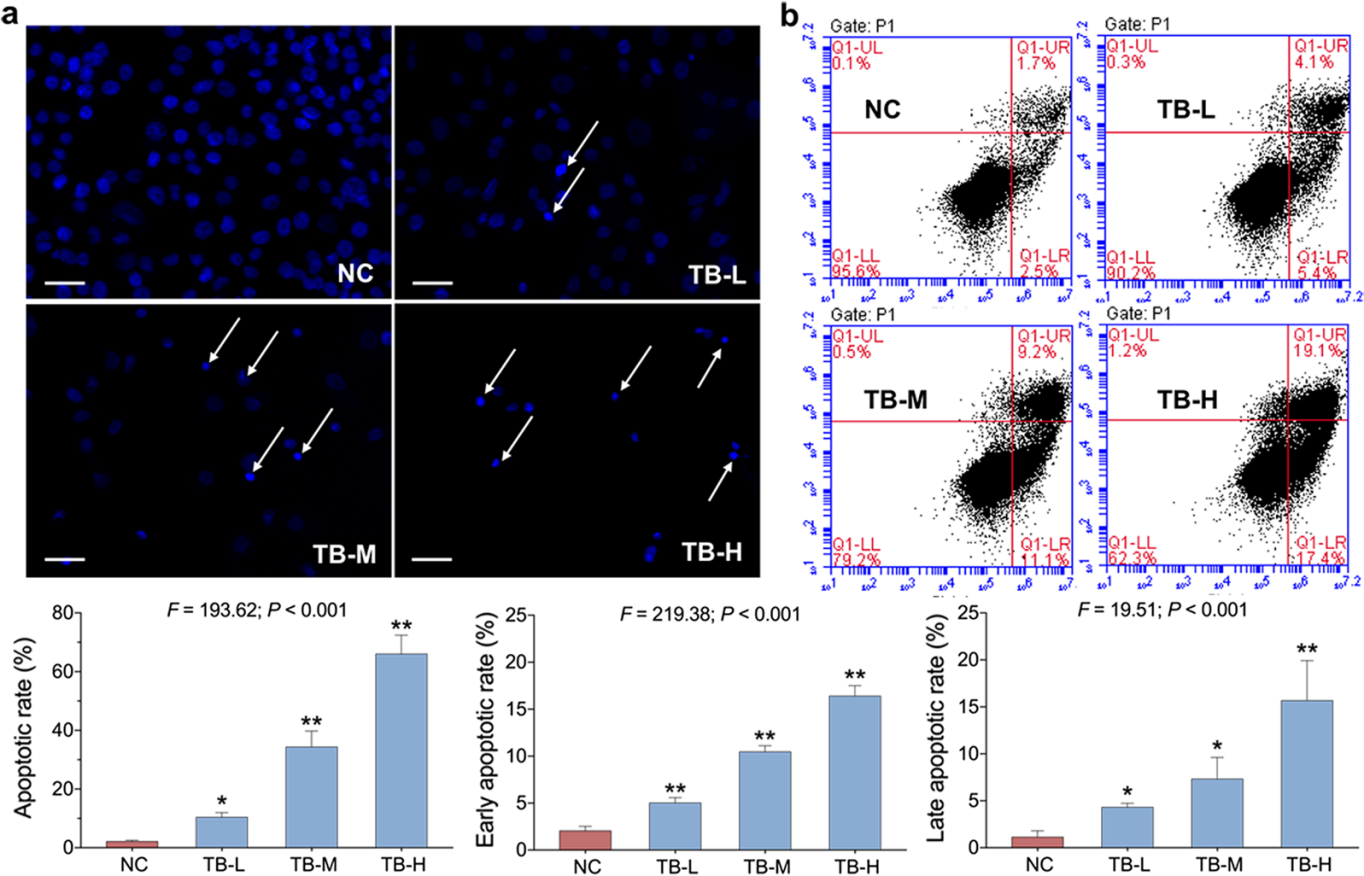
**a** Representative images of DAPI staining of SK-Hep-1 cells with TB treatment for 24 h. Scale bar: 50 μm. **b** Flow cytometry analysis on SK-Hep-1 cell apoptosis with TB treatment for 24 h. Values were presented as the mean ± SD (n = 3). *p < 0.05 and **p < 0.01 vs. normal control

DAPI staining was conducted to assess TB-induced apoptosis of SK-Hep-1 cells. As depicted in Fig. 3a, the untreated cells had rounded nuclei with normal blue color, whereas the TB-treated cells had bright blue nuclei and condensed chromatin with apoptotic bodies (indicated by arrows). Similarly, Annexin-V/PI staining also showed significant TB-induced apoptosis on SK-Hep-1 cells (Fig. 3b). The total apoptotic rates (early and late) of TB at 50, 100 and 150 µg/ml were 10.40%, 34.38% and 66.04%, respectively. These results suggested that TB dose-dependently induced apoptosis on SK-Hep-1 cells.

### Pro-senescent effect of TB

**Fig. 4:**
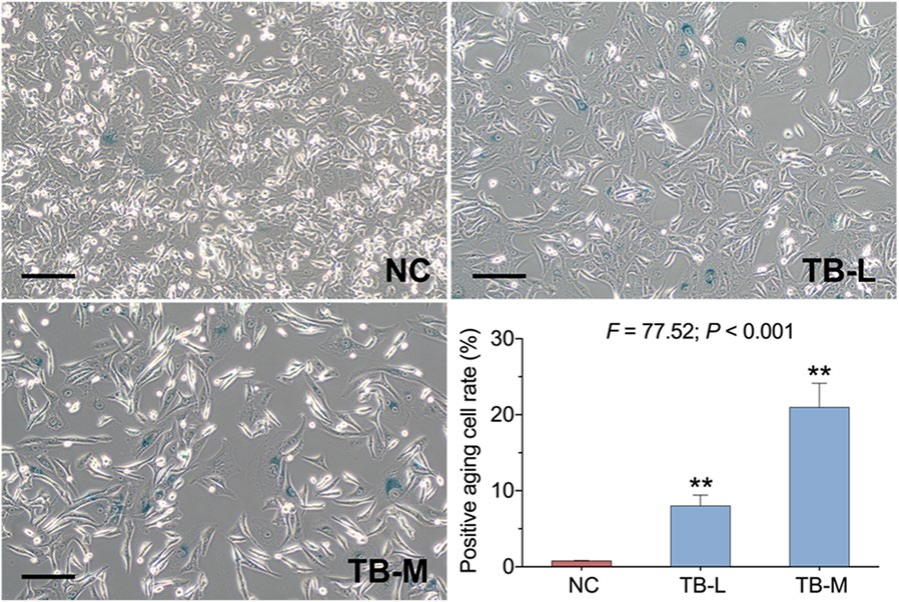
Representative images of SA-β-gal staining of SK-Hep-1 cells with TB treatment for 48 h. Scale bar: 200 μm. Values were presented as the mean ± SD (n =5). **p < 0.01. vs. normal control

SA-β-gal assay was conducted to evaluate the senescence induced by TB on SK-Hep-1 cells. As shown in Fig. 4, the number of SA-β-Gal-positive cells was obviously increased with TB treatment from 50 to 100 µg/ml, indicating that TB dose-dependently induced senescence on SK-Hep-1.

### Molecular actions of TB

**Fig. 5:**
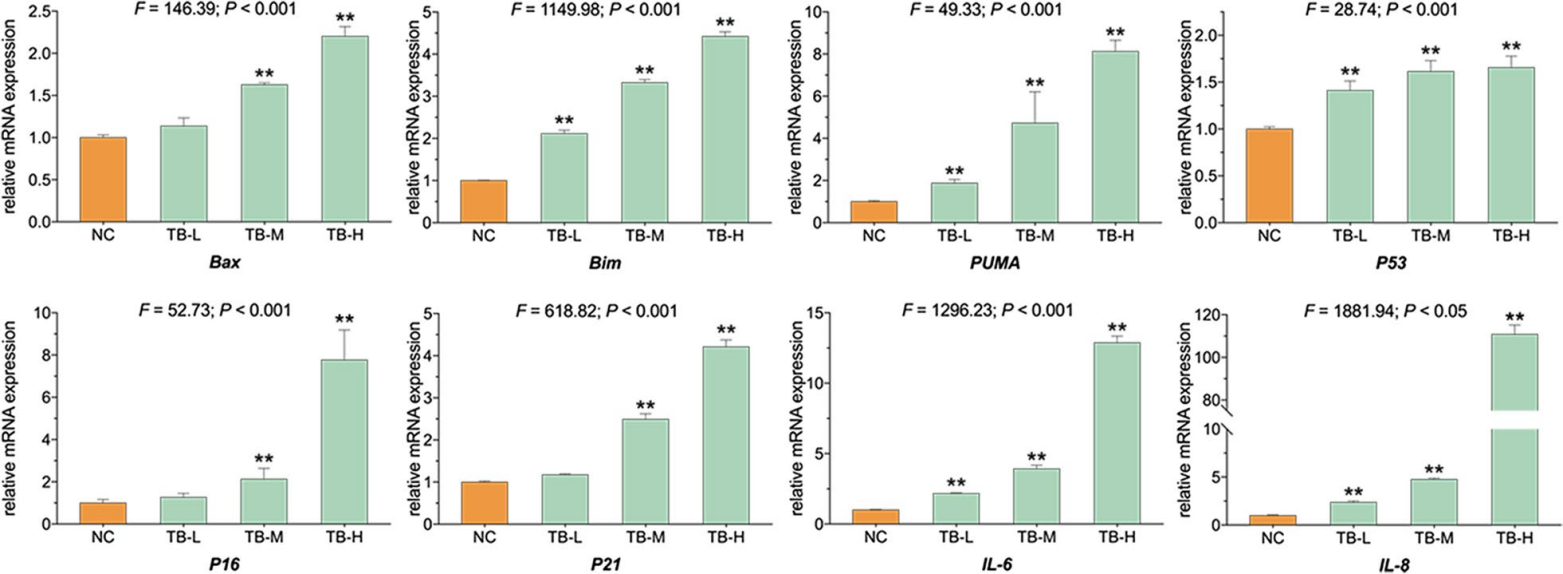
Relative mRNA expression of target genes in SK-Hep-1 cells with TB treatment for 24 h. Values are presented as mean ± SD (n = 3). **p < 0.01 vs. normal control

The relative mRNA expression of targeted genes in TB-treated SK-Hep-1 cells was determined by qPCR assay. As depicted in Fig. 5, the expression of *P53* was dose-dependently up-regulated by TB at the transcriptional level. TB also increased the expression of down-stream senescent genes (*P16*, *P21*, *IL-6* and *IL-8*) and apoptotic genes (*Bax*, *Bim* and *PUMA*).

**Fig. 6:**
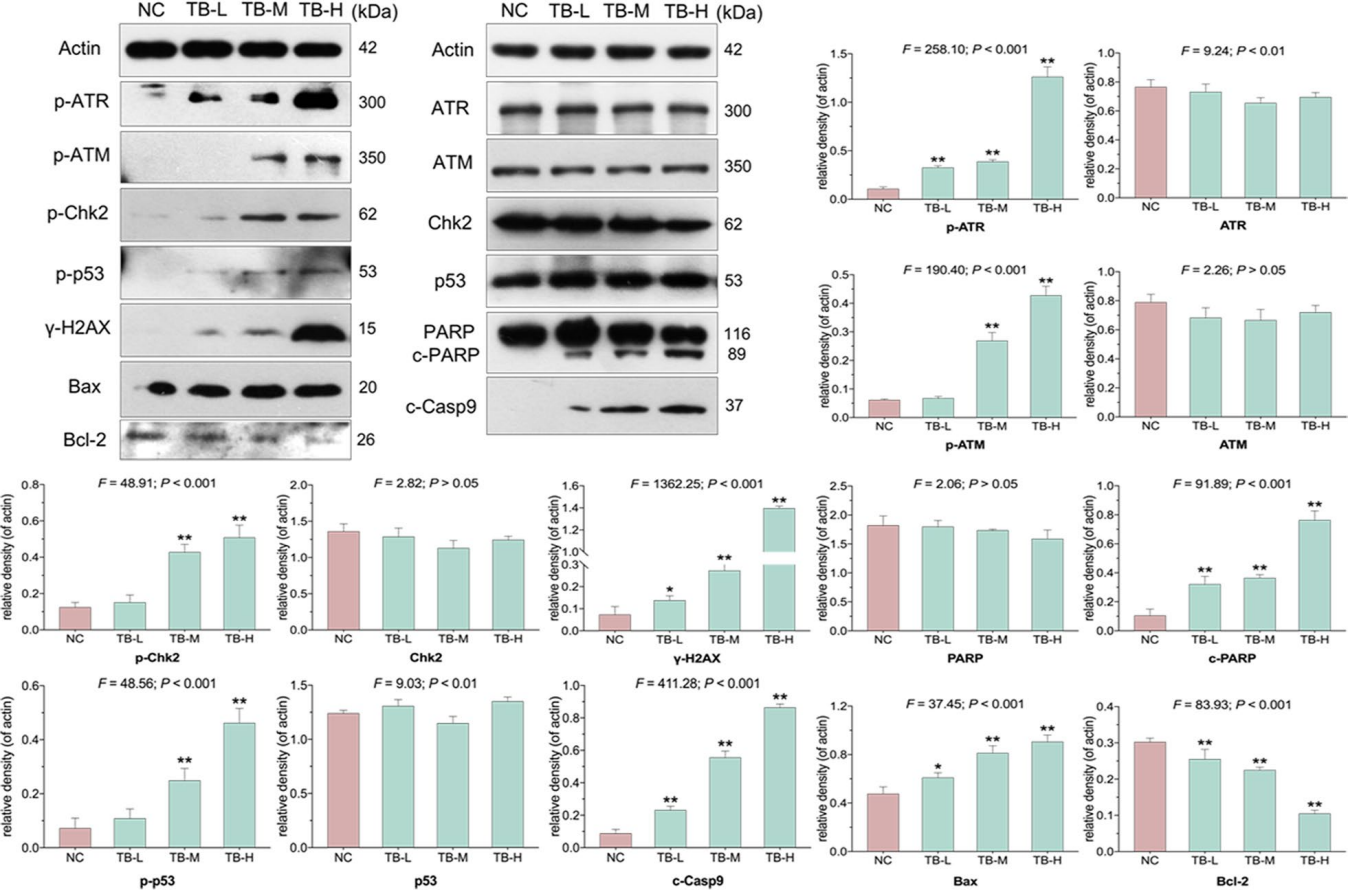
Expression and phosphorylation of targeted proteins in SK-Hep-1 cells with TB treatment for 24 h. Values represent mean ± SD (n = 3). *p < 0.05 and **p < 0.01 vs. normal control

The protein expression of targeted molecules in TB-treated SK-Hep-1 cells was determined by WB. A strong phosphorylation of ATM, ATR, Chk2, and p53 was detected in TB-treated SK-Hep-1 cells (Fig. 6), indicating the activation of p53 signaling pathway. From low to high doses, TB obviously increased the expression of downstream apoptotic markers (γ-H2AX, c-PARP, c-Casp9 and Bax ) with down-regulation of anti-apoptotic protein Bcl-2. The regulatory effects of TB on these genes and protein were dose-dependent.

### Verification of p53-mediated mechanism of TB

**Fig. 7:**
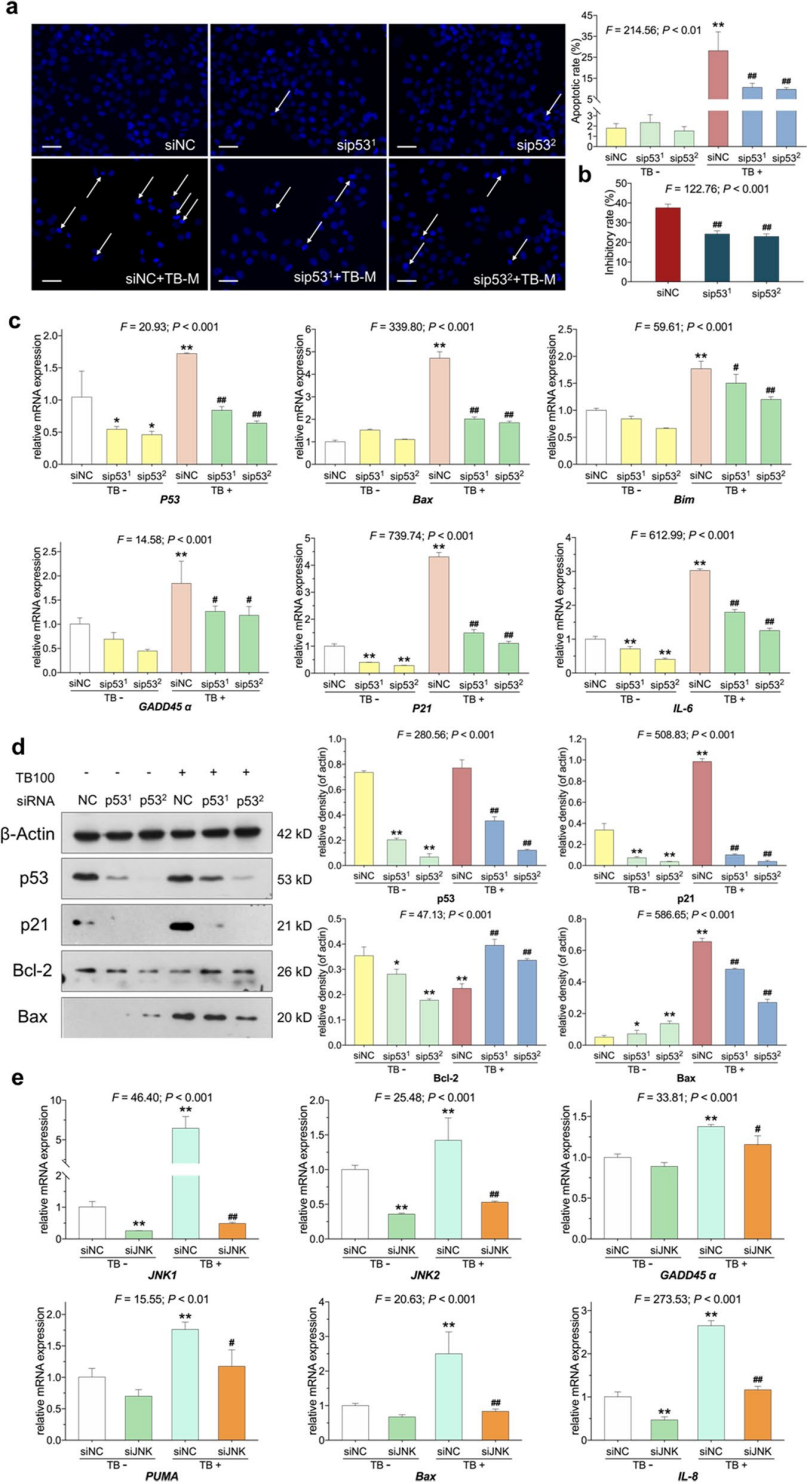
**a** Apoptotic morphology of SK-Hep-1 cells with p53-siRNA and TB (100 µg/ml) treatment by DAPI staining. Scale bar: 50 μm. Values are presented as mean ± SD (n = 5). **p < 0.01 vs. siNC group, ^##^*p* < 0.01 vs. siNC group plus TB treatment. **b** Inhibitory rate of TB (100 µg/ml) with nontargeting control siRNA or p53-siRNA treatments on SK-Hep-1 cells at 24 h. Values were presented as the mean ± SD (n = 5). ^##^*p* < 0.01 vs. siNC group plus TB treatment. **c** Relative mRNA expression of SK-Hep-1 cells with p53-siRNA and TB (100 µg/ml) treatments for 24 h. Values are presented as mean ± SD (n = 3). *p < 0.05 and **p < 0.01 vs. siNC group, ^#^*p* < 0.05 and ^##^*p* < 0.01 vs. siNC group plus TB treatment. **d** Relative protein expression of SK-Hep-1 cells with p53-siRNA and TB (100 µg/ml) treatments for 24 h. Values are presented as mean ± SD (n = 3). *p < 0.05 and **p < 0.01 vs. siNC group, ^##^*p* < 0.01 vs. siNC group plus TB treatment. **e** Relative mRNA expression of SK-Hep-1 cells with JNK-siRNA and TB (100 µg/ml) treatments for 24 h. Values are presented as mean ± SD (n = 3). *p < 0.05 and **p < 0.01 vs. siNC group, ^#^*p* < 0.05 and ^##^*p* < 0.01 vs. siNC group plus TB treatment. siNC, nontargeting control siRNA-treated group; sip53, p53-siRNA-treated group; siJNK, JNK-siRNA-treated group

p53-siRNA was applied to determine whether p53 signaling mediates the senescence and apoptosis in TB-treated SK-Hep-1 cells. The transfection efficacy was confirmed by qPCR and WB assays (Fig. 7c and d). Cell viability assay and DAPI staining showed that p53-siRNA significantly counteracted the TB-mediated anti-proliferative and pro-apoptotic effect on SK-Hep-1 cells (Fig. 7a and b). qPCR assays revealed that p53-siRNA significantly suppressed the expression of senescent genes (*P21*, *GADD45α* and *IL-6*) and antagonized the regulation of TB on these senescent genes and apoptotic genes (*Bax* and *Bim*) (Fig. 7c). WB assays revealed that p53-siRNA notably decreased the expression of p21 and Bax, increased the expression of Bcl-2, and counteracted the regulation of TB on these proteins.

Our previous studies have shown that TB induced apoptosis on p53-mut HCC cells via activation of JNK signaling [39]. Thus, JNK-siRNA was applied to verify the involvement of JNK in the action mechanism of TB. As shown in Fig. 7e, TB obviously up-regulated the expression of *JNK1* and *JNK2* and JNK-siRNA substantially reversed the regulation of TB on these genes as well as on the senescent (*GADD45α* and *IL-8*) and apoptotic genes (*Bax* and *PUMA*). The above results indicated that p53 signaling and JNK participated in the TB-induced cellular senescence and apoptosis of SK-Hep-1 cells.

## Discussion

TB is known to improve the lipid metabolism and reduce the cholesterol level of liver, indicating its beneficial effect on liver [34]. Our study revealed that TB exhibited significant inhibitory effects on HCC cell lines (SK-Hep-1, HepG2, and Huh7), in which the effect on p53-WT SK-Hep-1 cells was the strongest. HepG2 cells are well-differentiated, which can well defend cellular stresses. In comparison, SK-Hep-1 cells were poorly differentiated with more sensitivity to cellular stresses, and easily suffered from severer DNA strand breaks [40]. Thus, SK-Hep-1 cells might be more sensitive to the treatment of TB, which explained why the effect of TB on HepG2 cells was milder than SK-Hep-1. Accordingly, we conducted the present study to determine the anti-HCC efficacy and explore the mechanism of TB on SK-Hep-1 cell line. Our findings demonstrated that TB significantly suppressed SK-Hep-1 tumor growth in xenograft zebrafish. Zebrafish, an important model for cancer research, overcomes the drawbacks of murine xenograft models as follow: (1) no immune rejection at larval stage; (2) transparent body enabling live imaging of tumor growth; and (3) lower ethical impact when used at the larval stage [41]. Surprisingly, we found that Cis-platin showed slightly weaker inhibitory effect than high dose of TB. This maybe because oral administration of Cis-platin through cultured water was performed instead of the routine intravenous injection, which might reduce its bio-availability. Interestingly, the reported in vitro IC_50_ of Cis-platin (5.13 ± 0.09 µg/ml) was much lower than that of TB [42], suggesting a difference of in vivo mechanism between TB and Cis-platin. The effective dose range of TB (from 1.7 to 16.7 µg/ml) in zebrafish can be estimated as 0.08 to 0.80 mg/kg in human by dose conversion, and dosage of green tea in human was roughly 1.13 to 13.17 mg/kg, which are very low and suitable for clinical application [43, 44]. In vitro results showed that TB induced cellular senescence and apoptosis of SK-Hep-1 cells through activation of ATM-Chk2-p53 cascade with bypass regulation of JNK. The innovation points of this study are as follows: (1) demonstration of the inhibitory efficacy of TB on p53-WT HCC cells (SK-Hep-1); (2) clarification of the action mechanism of TB through cell apoptosis and cellular senescence; and (3) determination of TBʹs molecular mechanism via ATM-Chk2-p53 signaling pathway with JNK bypass regulation.

Cellular senescence is a cell state characterized by permanent cell-cycle arrest with widespread changes in chromatin organization and gene expression [45, 46]. p53 and pRb are critical transcriptional regulators in cellular senescence. p21 is one of the most important targets of p53 transcriptional activity in senescent process, whereas p16 is a positive upstream regulator of pRB [47]. A vital feature of senescent cells is the secretion of senescent associated secretory phenotype (SASP), such as pro-inflammatory cytokines and chemokines, growth factors, etc. [48, 49]. Except for p53, p16, and p21, IL6 and IL8 are also the central components of SASP and act as important markers of cellular senescence [50, 51]. Our data showed that TB significantly induced senescent phenotype (SA-β-gal positive) of SK-Hep-1 cells with up-regulation of all the above senescent markers (*P53*, *P16*, *P21*, *IL-6* and *IL-8*) (Figs. 4 and 5), indicating that TB induced cellular senescence to suppress SK-Hep-1 cells through p53-related mechanism. Some chemotherapeutics possess anti-cancer efficacy via inducing cellular senescence, such as Palbociclib, a specific CDK4/6 inhibitor, which was approved in 2015 for clinical treatment of advanced breast cancer [52].

**Fig. 8:**
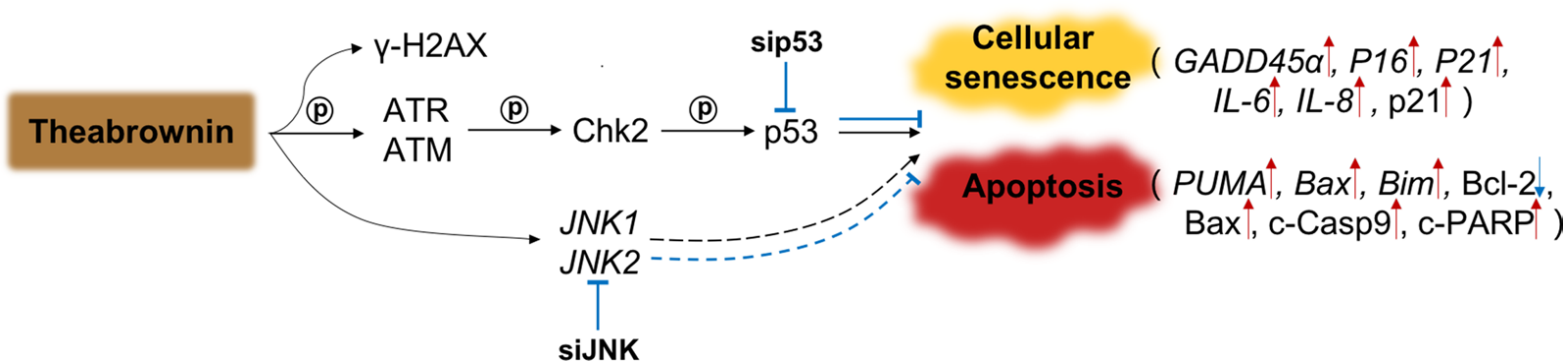
Schematic diagram of the mechanism of TB on SK-Hep-1 cells

Besides the cellular senescence, TB meanwhile induced apoptosis of SK-Hep-1 cells. The pro-apoptotic p53 signaling pathway was activated by TB through activation of p-ATM, p-ATR, γ-H2AX, p-Chk2, and p-p53 (Fig. 6). ATM and ATR are initiating kinases of DNA damage response (DDR) cascade, while γ-H2AX serves as a sensitive biomarker for DNA damage during DNA double-strand breaks (DSBs) [53, 54]. In response to the DNA DSBs, ATM phosphorylates itself at Ser1981 to activate extensive substrates to mediate cell cycle checkpoint control, DNA repair or apoptosis [55]. The serine/threonine kinase Chk2 is another component of DDR, which requires ATM-activated phosphorylation at several residues including Thr68 [56]. Activated Chk2 phosphorylates p53, enhancing its stability and activity to induce apoptosis through Bcl-2 and caspase-dependent manners [57, 58]. Therefore, TB induced apoptosis of SK-Hep-1 cells with DNA DSBs through ATM-Chk2-p53 signaling pathway. Subsequently, the downstream apoptotic proteins (Bax, c-Casp9 and c-PARP) and genes (*PUMA, Bim* and *Bax*) were activated, resulting in the mitochondrial pathway of apoptosis (Fig. 8). Casp9 is the initiating caspase associated with the mitochondrial apoptosis [59]. Once activated, Casp9 cleaves and activates downstream effectors to cleave PARP, which promotes cellular disassembly and serves as a hallmark of apoptosis [60, 61].

Previous report has shown that p53 signaling plays a critical role in modulating cellular responses to DNA damage, leading to irreversible cellular senescence and apoptosis [62]. This study verified the p53 signaling-mediated pro-apoptotic and pro-senescent mechanism of TB by using p53-siRNA (Fig. 7). In our previous study, TB induced apoptosis in p53-mut Huh7 cells via activation of JNK signaling pathway, indicating that JNK might also participate into the action mechanism of TB on HCC cells [39]. Correspondingly, we revealed that JNK was a bypass regulator involved in the mechanism of TB (Fig. 7e), suggesting a multi-target mechanism of TB on both ATM-Chk2-p53 and JNK signaling (Fig. 8).

Alike most natural products, TBʹs in vitro effects on cancer cells are milder than chemotherapeutics, with higher IC_50_s. However, this study demonstrated that TB had considerable efficacy against HCC cell mass in xenograft zebrafish and the in vivo efficacy was even better than Cis-platin. Our finding was in consistent with previous reports about the in vivo anti-cancer study of TB [37, 39], which suggested that TB might not only induce cancer cell apoptosis in a direct way, but also suppress in vivo cancer cell mass in an indirect way (e.g., anti-angiogenesis and immunoregulation) [63–65]. Further studies are needed to explore the in vivo mechanism of TB.

## Data Availability

All data generated or analyzed during this study are included in this published article and its supplementary information files.
